# The neuroinflammation collection: a vision for expanding neuro-immune crosstalk in *Brain*

**DOI:** 10.1093/brain/awab187

**Published:** 2021-05-13

**Authors:** Sarosh R Irani, Avindra Nath, Frauke Zipp

**Affiliations:** 1 Oxford Autoimmune Neurology Group, Nuffield Department of Clinical Neurosciences, John Radcliffe Hospital, University of Oxford, Oxford, UK; 2 Department of Neurology, Oxford University Hospital, NHS Foundation Trust, Oxford, UK; 3 Section of Infections of the Nervous System, National Institute of Neurological Disorders and Stroke, National Institutes of Health, Bethesda, MD 20892, USA; 4 Department of Neurology, Focus Program Translational Neuroscience (FTN) and Immunotherapy (FZI), Rhine Main Neuroscience Network, Johannes Gutenberg University Medical Center Mainz, Germany

The study of neuroinflammation provides an exciting opportunity to rapidly understand therapeutically amenable components of many illnesses embedded within the core practices of neurology and psychiatry. In addition to crucial therapeutic benefits, advances in understanding the immune basis of primary autoimmune conditions can present key aetiological and diagnostic insights. Recent advances have led to the growing appreciation for a role of the immune system in the pathogenesis of many other conditions, considered likely to have non-immune primary drivers (Fig. 1). These clinical arenas span neurodegenerative and neuro-infectious conditions in addition to neuro-oncology, neuropsychiatry, stroke and traumatic brain injury. 

**Figure 1:**
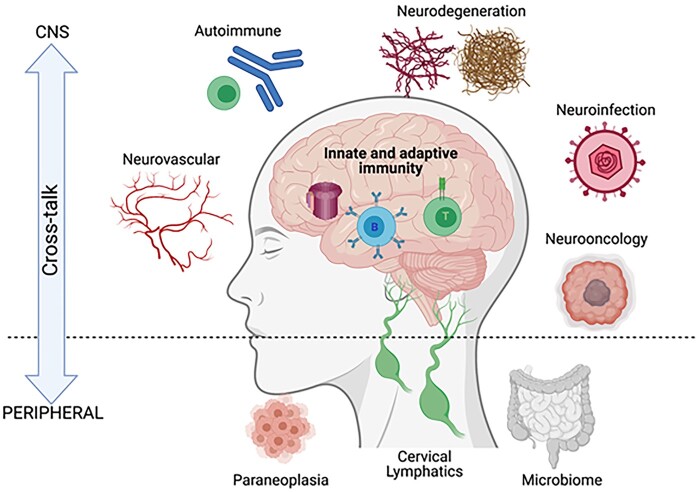
**Pathological and homeostatic neuroimmune interactions in the peripheral and central nervous system.** Innate and adaptive immunity operate in a variety of pathological CNS states, with peripheral contributions from systemic tumours (paraneoplasia), cervical lymphatics (believed to drain meningeal lymphatics) and the gut microbiome. Created in BioRender.

In all these, potential immune contributions offer therapeutically tractable approaches. From a biological perspective, a more comprehensive understanding of clinically relevant neuroimmune interactions naturally segues into important basic questions about the anatomy and function of connections between the periphery and the CNS (Fig. 1), and the relative roles of immune system components in mediating these effects. While these questions should be studied in both health and disease, many observations arise most starkly when applying mechanisms of homeostasis in the context of pertinent diseases.

Herein, we highlight eight top neuroinflammation publications in *Brain* between 2020 and 2021 and use these as a focal point to offer a vision of how anticipated developments in neuroinflammation are likely to impact our understanding of key elements of neurology, and lead to new treatments. In addition, this vision will provide a framework for a series of review articles to stimulate ideas and highlight some of the most exciting areas within this rapidly expanding, clinically relevant and highly translational field.

## Prototypical immune-mediated conditions

The autoantibody-mediated diseases of the nervous system provide perhaps the most direct examples to connect an identified pathogenic agent with the corresponding disease process. In these conditions—ranging from myasthenia gravis and neuromyelitis optica to autoimmune encephalitis—autoantibodies target extracellular domains of neuroglial proteins and patients often show highly distinctive, even unique, clinical features. In autoimmune encephalitis, these include faciobrachial dystonic seizures and multiple frequent focal seizure semiologies in patients with leucine-rich glioma-inactivated 1 (LGI1) antibodies,^1^ while a stereotyped multistage progression of inherently complex clinical features characterizes the phenotype of individuals with *N*-methyl-d-aspartate receptor (NMDAR) antibody encephalitis.^2^^,^^3^ In these patients, the highly characteristic nature of these—and other—clinical features provides persuasive evidence of a distinctive human pathology directly mediated by these antibodies. Further to these clinical observations, experimental data confirm these autoantibodies fulfil modified Koch’s postulates for pathogenicity, both *in vitro* and in animal models.^4^^,^^5^ Hence, a logical prediction is that inhibiting their end-target effects will reverse symptoms in patients.

To test this hypothesis at the molecular level, an allosteric modulator of the NMDAR—which crosses the blood–brain barrier—was applied to culture and *in vivo* model systems prior to application of patient NMDAR antibodies.^6^ Administration of this drug to mice subsequently receiving a cerebroventricular transfer of NMDAR antibodies prevented all the antibody-mediated effects: memory alterations, a spatial redistribution of NMDARs and the abrogation of long-term potentiation. Yet, intriguingly, this drug did not fully abrogate NMDAR internalization, which is often characterized as the dominant mechanism of action of the human autoantibodies. However, this study lacked a key experiment: namely the application of the drug after the induction of antibody-mediated pathology. Hence, its clinical relevance as a symptomatic therapy, alongside the observationally established value of immunotherapies, remains a key area for future work.

Indeed, it is possible that in a patient in whom relentless autoantibody production is ongoing, efforts to block end effector autoantibody-mediated mechanisms of brain dysfunction prove fruitless. In contrast, the underlying immunology may present greater therapeutic value. Furthermore, in autoantibody-mediated diseases, working backwards from the soluble autoantibodies to the autoantigen-specific B and T cells and, ultimately, the auto-immunizing events, should provide a far more comprehensive account of disease pathogenesis. Yet, despite the multipronged importance of the immunobiology, few studies have addressed this aspect.

A recent paper in *Brain* isolated a suite of LGI1-specific B cells from patients and retrieved individual LGI1-reactive immunoglobulins to characterize aspects of the disease immunology and neuroscience, in parallel.^7^ The immunology inferred from these B cells revealed somatic hypermutation of highly diverse immunoglobulin species, suggesting their derivation from a classical, polyclonal, peripheral germinal centre response. The neuroscience revealed pathogenic effects—both *in vitro* and *in vivo*—with a marked dependence on the targeted epitopes and individual antibody affinities. Hence, the results of this study suggested that the precise nature of the matured autoimmune response could determine its potential pathogenicity.

## Neurodegeneration

In neurodegenerative diseases, dominant theories concern the necessary and sufficient nature of toxic aggregated proteins as directly pathogenic agents. Removal of these neuroactive species in patients has harnessed a major supremo of immunology: the monoclonal antibody. For example, an amyloid-directed monoclonal antibody effectively removed plaques in humans with Alzheimer’s disease.^8^ However, there was no associated clinical improvement. In part due to this high-profile clinical inefficacy, the more therapeutically amenable immune system has attracted much attention across neurodegeneration. While a primary immune pathogenesis remains unlikely in neurodegeneration, mounting evidence suggests early potential contributory roles for several limbs of the immune system.

A recent article in *Brain* studied over 100 Down syndrome brain specimens and cultured primary neurons from individuals between 16 gestational weeks and 64 years old.^9^ This remarkable collection of specimens identified activated microglia (the key innate phagocytic cell resident to the CNS) and inflammatory cytokines in young adults with Down syndrome. Importantly, these observations were made at time points coincident with only sparse histological evidence of Alzheimer’s pathology. Also, older Down syndrome adults showed alternative cytokine profiles. Hence, the authors suggested early anti-inflammatory therapies should be explored in human Alzheimer’s disease. Down syndrome cases may represent an important pre-amyloid human model for testing this hypothesis.

While attention regarding neuroinflammation in neurodegeneration has centred on innate immune mechanisms, a few reports have provided additional insights by studying the antigen-experienced acquired limb of immunity. Hence, a *Brain* paper describing a robust CD8^+^ T cell infiltration into the substantia nigra pars compacta of Parkinson’s disease brains was a major breakthrough.^10^ These T cells expressed directly cytolytic factors, including granzymes (serine proteases released by cytoplasmic granules), in addition to pro-inflammatory cytokines. Furthermore, a potentially pathogenic role for their observed direct contact with dopaminergic neurons was strengthened by higher CD8^+^ densities in samples with fewer dopaminergic neurons. Perhaps most importantly, these observations were apparent prior to the accumulation of α-synuclein, supporting the author’s bold suggestion that the immune system may ‘initiate and propagate neuronal death and synucleinopathy in Parkinson’s disease’. Together with the identification of antigen-specific T cells and HLA variants in Parkinson’s disease,^11^ these collective findings create a foundation for trials of immune tolerizing agents in patients with this and other forms of neurodegeneration.

## Multiple sclerosis and CNS neoplasia

**Figure 2:**
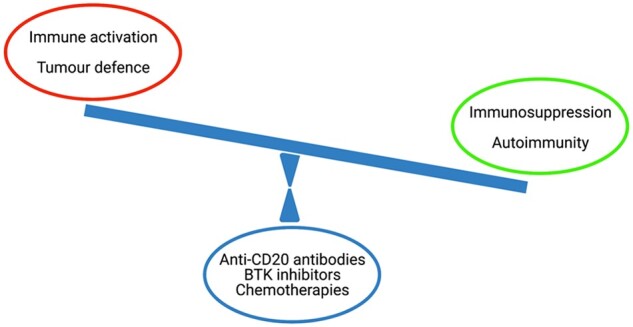
**The delicate balance of autoimmunity and neoplasia in neuroimmunology.** The efficient induction and sustainment of immune responses are prerequisites to control malignant transformation (high level of immune activation, red circle), whereas autoimmune disorders require successful anti-inflammatory therapy (dampening immune activation, green circle). Only when the immune system itself is malignantly transformed therapeutic strategies point in the same direction targeting neoplasia and autoimmunity (blue circle).

The varied facets of crosstalk between immune and nervous systems are clear when attempting to understand or treat autoimmunity and neoplasia. These carry inherent challenges due to the yin-yang of necessary immunosuppression in inflammatory conditions and obligatory immune stimulation for tumour defence. The latter provokes autoimmunity,^12^^,^^13^ whereas blocking immune cells from entering the brain in order to effectively treat autoimmunity is closely associated with a potentially devastating adverse event, namely progressive multifocal leukoencephalopathy (PML). Only if the immune system itself is tumorous (e.g. lymphoma) or in the case of a non-selective treatment strategy with cell toxicity, can therapy be the same for both cancer and autoimmunity (Fig. 2).

This double-edged sword of immune mechanisms may not be the only enigma when considering the lack of successes in treating glioblastoma. Recent exciting discoveries suggest neuronal properties of glioblastoma tissue.^14^^,^^15^ Moreover, the neurotransmitter glutamate was found to be released by rodent and human T helper 17 cells, which seems to exclude this arm of immune defence for treatment concepts of glioblastoma.^16^ To date it is not known how glioblastoma so effectively evade immune defences. A paper in *Brain* offers a combination of different mechanisms leading to lowered immunity not exclusive to brain tumours, but present in several acute neurological insults and thus possibly specific for the CNS.^17^ This concept needs proof in patients. The urgent clinical need for effective treatments for brain tumours and metastases together with completely novel research directions both underline the utmost importance of advancing research in such aggressive conditions of the CNS.

Despite several decades of research, the field of multiple sclerosis, the most frequent classical autoimmune disorder of the CNS potentially leading to devastating disability in young adults, on the one hand regularly delivers exciting novelties, but on the other still lacks full clarification of the pathology as well as sufficient evidence for successful therapies. Moreover, it is still unclear how the disease is initiated or why grey matter damage patterns—most relevant for disability—seem to differ in different periods of the disease and across brain/spinal cord regions. Unexpected outcomes of treatment trials have revealed that, for example, B cells and cytokines (including IL-17 and TNF-alpha) serve roles other than those originally identified—also pointing to difficulties of ‘true’ translation dogmatically controversial within the community. However, technical advances in immunological and neuroscientific methodologies have led to significant progress and regularly unearth unexpected findings. One recent example is the possibility to detect the extent of neuronal injury outside of the brain, via the assessment of neurofilament light chain (NfL) levels in blood. The value of NfL for clinical progress, namely supporting prognostication and therapy selection, is just one example of stratification, which might benefit both patients and physicians in the near future.

After great efforts, the first therapy for primary progressive multiple sclerosis tackling grey matter pathology was achieved by B cell depletion. Bringing different research angles together, a recent experimental study published in this journal demonstrates that novel Bruton tyrosine kinase (BTK) inhibitors, which target B cells, in fact reduce leptomeningeal lymphoid follicles thought to be a feature of progressive multiple sclerosis.^18^ The latter are suspected to be involved in grey matter pathology, bringing the recently appreciated role of B cells—as well as meningeal immunity—into the spotlight.

Nevertheless, it is surprising how many open questions remain. Given the intensive research and advances in the gut-brain axis, including the influence of nutrition and environment; novel roles for old players, such as astrocytes; and significant developments regarding lymphatics, glymphatics and CNS barriers. Specific points that will need to be clarified include:

(i) Why are current therapeutics more effective if they are commenced in the first few years after disease onset?
(ii) Why are they less effective in older people?
(iii) How can progression be tackled more efficiently than by depleting immune cell populations?
(iv) What happens to beneficial roles of the immune system in the CNS upon immunosuppressive or immunomodulatory therapy?
(v) Should neural precursor cells still be pursued as a therapeutic option?
(vi) Given the disappointment with negative anti-LINGO antibody trials, is remyelination still a possible treatment goal?
(vii) Are there unidentified roles of the immune/nervous system crosstalk beyond what is known and what has been indicated by the large genetic map developed by the International MS Genetics Consortium IMSGC?
(viii) Which experimental successes—such as the intrathecal mesenchymal cellular approach highlighted in this journal^19^—can be expected to fulfil requirements for use in human (e.g. reach the human CNS) and thus be transferred to patients?

## Inflammation in cerebrovascular disease

We have made enormous strides in treating acute stroke, however, the subacute phase where there are cellular infiltrates and inflammation still remains a challenge. Understanding the pathophysiology in humans and appropriate animal models such as non-human primates or other animals that have chronic risk factors similar to patients with stroke could help advance the field by identifying new modes of intervention. Although the potential role of inflammation in mediating atherosclerotic injury and cerebrovascular disease has long been debated, the relationship between the two remains unclear.

One way to address this might be to determine the pattern and magnitude of cerebrovascular disease in patients with known inflammatory disorders of the CNS. In a study published in this journal, the authors characterized the brain and systemic vascular pathology in the arterial system in patients with multiple sclerosis at autopsy.^20^ They found that even though arterial disease can be found in patients with multiple sclerosis, they appear to be independently regulated without a common pathogenic mechanism. These findings raise several important questions regarding the antigenic targets of the arterial and venous inflammatory processes in multiple sclerosis. Further, it may stimulate interest in whether current disease-modifying therapies can also affect the inflammatory processes within the vasculature.

## Harnessing innate immune responses in the brain

Stimulation of innate immune responses is considered detrimental to the brain and have been implicated in the pathophysiology of several, if not most, chronic neuroinflammatory, neuroinfectious and neurodegenerative diseases.^21^ Therapeutic approaches under consideration include strategies to target cytokines, glial cell activation and complement pathways. While a large body of experimental data supports this approach, careful consideration is necessary when blocking host immune responses, for it may be another double-edged sword. For example, despite strong experimental evidence supporting tumour necrosis factor-alpha as a causative factor in myelin injury in multiple sclerosis, a clinical trial with anti-tumour necrosis factor-alpha agents precipitated acute attacks of the disease.^22^

By analogy, a study recently published in this journal showed that the gamma subunit of complement component 8 (C8G) inhibits neuroinflammation.^23^ This is a surprising finding since the majority of complement components are proinflammatory and often exert widespread effects. However, Kim *et al*.^23^ found that C8G was expressed in astrocytes upon activation by proinflammatory cytokines released by activated microglia. The neuro-specificity of C8G was shown via its potent inhibition of the inflammasome of microglia and the authors suggested that C8G could potentially be used as a therapeutic agent in neuroinflammatory and neurodegenerative diseases.

## Relatively neglected areas: infection, psychiatry and others

There is a close relationship between microbial pathogens and inflammation. The immune system is the major defence against these organisms. A significant immune response can result in clinical manifestations from an inflammatory syndrome such that the organism itself may get masked and hard to detect. This is most apparent in the brain where even small amounts of inflammation can be symptomatic. For these reasons, viruses have long been suspected to be the aetiological agent for many neuroimmune and neurodegenerative diseases but this has been hard to prove. For example, over the years, several viruses have been implicated as the aetiology of multiple sclerosis, including canine distemper virus, Epstein Barr virus, human herpesvirus 6, retroviruses and others but none has been proven as of yet. Similarly, herpes viruses have been implicated in Alzheimer’s disease and retroviruses in amyotrophic lateral sclerosis. These relationships are active areas of investigation and major efforts are underway to determine the role of these agents in the pathophysiology of several neurological diseases.

All the same, it is clear that under some circumstances, viral infections can trigger well known autoimmune diseases. For example, herpes simplex virus type 1 encephalitis can result in an autoimmune encephalitis by generation of autoantibodies to neuronal receptors.^24^ Similarly, a parasitic infection, onchocerciases, has been shown to trigger autoantibodies against leomodin-1 resulting in a distinct epilepsy called Nodding syndrome.^25^ Once these autoimmune syndromes have been triggered they may be self-perpetuating even if the organism itself has been adequately treated.

These virus-triggered immune syndromes or post-viral syndromes have never been more apparent than what we are facing today in the most extraordinary and unprecedented times of the SARS-CoV-2 infection. This virus, similar to other coronaviruses, rarely causes encephalitis yet it can associate with several neuroimmune disorders such as acute disseminated encephalomyelitis, acute necrotizing haemorrhagic encephalopathy, microvascular disease with antiphospholipid syndrome, transverse myelitis, myositis and Miller-Fisher syndrome.^26^ A potential association between Guillain-Barré syndrome and SARS-CoV-2 remains controversial.^27^ In contrast, Zika virus has been strongly associated with Guillain-Barré syndrome.^28^ In children, SARS-CoV-2 can cause a multi-system inflammatory syndrome that also affects the brain.^29^

Of major concern is that many patients who developed mild symptoms during the initial phase of the pandemic are now complaining of multiple neurological symptoms such as exercise intolerance, dysautonomia, cognitive dysfunction and pain syndromes. The possibility has been raised that these patients may not have cleared the virus due to a lack of a robust immune response in the initial phase resulting in persistent or restricted viral.^30^ More extreme examples of this phenomenon have been described with other viruses, such as measles, which can cause subacute sclerosing panencephalitis several months or years after the initial infection. In these cases, the virus acquires mutations in the matrix and fusion proteins such that fully replicating viral particles cannot be formed and thus is not detected in the CSF or blood but it spreads in the brain by cell-to-cell contact. A similar phenomenon has been described with Dengue virus.^31^ The possibility that restricted viral replication may underlie other neuroinflammatory diseases needs to be explored. Viruses may also cross the placental barrier and result in congenital syndromes by affecting brain development. Classical examples include Zika virus and congenital rubella syndrome, where the virus infects neural progenitor cells and arrests neural development.^32^ The possibility that some of the other congenital malformations may be due to viral infections remains unknown.

A prevailing concept is that all microorganisms invade humans from the external environment. However, a large part of the human genome contains relics of prior viral infections that were incorporated over the process of evolution and then, over millions of years, have made multiple copies of themselves within the genome and acquired many mutations along the way. These retroviral elements have acquired important physiological functions in early embryonic development but are epigenetically silenced in adults. Reactivation of these viral elements have been described in various cancers and have recently been associated with neuroinflammatory and neurodegenerative diseases.^33^ Further investigations are needed to explore their pathophysiological relationships. *Brain* aims to publish updates and review articles on some of these topics and invites researchers to submit manuscripts that explore relationships between the microbes and human nervous system disease.

Despite rapidly emerging insights into the mutual interaction of the immune and the nervous systems, in particular within the CNS, several rarer disorders, like sarcoidosis and cerebral vasculitis, remain ‘under-researched’ areas, often without sufficient mechanistic and investigational precision. The same is currently true for post-infectious disorders including so-called ‘long COVID syndrome’.

An area of key interest, and with potential for major public health benefits, surrounds the contributions of autoimmunity and inflammation in primary psychiatric conditions. Within these disorders, the emerging concepts of immune-dependent brain homeostasis or resilience may exert major influences.^34^ Overall, the field of psychoneuroimmunology has observed a number of historical false dawns. More recently, several lines of data suggest that the study of immunity in these diseases can provide therapeutic hypothesis to test directly in humans. Observational data have implicated a variety of potentially inflammatory biomarkers which may offer pathogenic insights and aid patient stratification for immunotherapy trials, both in mood disorders and psychosis.^34-36^ Ultimate proof will likely require a positive result in such a trial. At *Brain* we are keen to publish methodologically sound clinical trials that offer insights into disease pathogenesis.

## Conclusions

Neuroinflammation is a rapidly evolving field with the realization that inflammatory cascades play an important role in the pathogenesis of several neurological diseases. However, the inflammatory response is complex and the cell types and signalling pathways involved may be disease-specific. Further, neuroinflammation can be both protective and detrimental to the host. Hence, a thorough understanding of these mechanisms and an open approach to both basic experimental and human study strategies is critical for development of optimal therapeutic strategies. We have highlighted several manuscripts published by *Brain* in the recent past focused on neuroinflammation. We invite authors to submit manuscripts in related areas that study human populations or experimental models and further our knowledge of these complex and emerging neuroimmune interactions.
